# Defining Ourselves: Personal Bioinformation as a Tool of Narrative Self-Conception

**DOI:** 10.1007/s11673-015-9690-0

**Published:** 2016-01-22

**Authors:** Emily Postan

**Affiliations:** Edinburgh Law School, The University of Edinburgh, Old College, South Bridge, Edinburgh, EH8 9YL UK

**Keywords:** Access to information, Biodata, Genetic origins, Narrative identity, Personal utility, Psychiatric neuroimaging

## Abstract

Where ethical or regulatory questions arise about an individual’s interests in accessing bioinformation about herself (such as findings from screening or health research), the value of this information has traditionally been construed in terms of its clinical utility. It is increasingly argued, however, that the “personal utility” of findings should also be taken into account. This article characterizes one particular aspect of personal utility: that derived from the role of personal bioinformation in identity construction. The suggestion that some kinds of information are relevant to identity is not in itself new. However, the account outlined here seeks to advance the debate by proposing a conception of the relationship between bioinformation and identity that does not depend on essentialist assumptions and applies beyond the narrow genetic contexts in which identity is customarily invoked. The proposal is that the identity-value of personal bioinformation may be understood in terms of its instrumental role in the construction of our narrative identities, specifically that its value lies in helping us to develop self-narratives that support us in navigating our embodied existences. I argue that this narrative conception provides useful insights that are pertinent to the ethical governance of personal bioinformation. It illuminates a wider range of ethical considerations in relation to information access; it accounts for variations in the utility of different kinds of information; and it highlights that the context in which information is conveyed can be as important as whether it is disclosed at all. These arguments are illustrated using an example drawn from psychiatric neuroimaging research.

## Introduction: Characterizing a Species of Personal Utility

This paper considers the nature of individuals’ interests in accessing “biological” information about themselves of the kind that is generated in healthcare, health-related research, screening, or testing services. This includes information about an individual’s past or present physical or mental health, and possible future health risks, and also extends beyond health, for example, to her bodily constitution and functions, reproductive or cognitive capacities, and biological relationships to others, including genetic relatedness or shared traits. For the purpose of this discussion these will be collectively termed “personal bioinformation,” signalling that they tell an individual something about herself, though this is not intended to preclude the shared nature of much of this information or others’ legitimate interests in it.

Practical guidance governing the disclosure of this kind of information to patients or participants in healthcare and health research contexts tends to focus on its clinical utility; that is, whether the information is sufficiently analytically robust and relevant to the individual to permit her, or her clinician, to make meaningful, actionable decisions about her health and care. For example, the precision of tests and availability of effective interventions underlie the U.K. guidelines governing the provision of screening programmes (Public Health England 2015), and the clinical significance of incidental findings has been a central consideration in U.K. Biobank’s deliberations about the ethics of providing participants with feedback from its new programme collecting imaging data (U.K. Biobank Ethics and Governance Council 2013, 8). However, it is increasingly common in the academic literature, especially that offering recommendations for ethical practice in genetic research or the regulation of genomic testing services, to encounter suggestions that there may be justification for taking into account the “personal utility” or “personal meaning” of findings in determining policies for their disclosure (Bunnik, Janssens, and Schermer 2015; Fabsitz et al. 2010; Khoury et al. 2010; Wolf et al. 2012). What is less clear, however, is precisely what the nature of personal utility is and why it is worth taking seriously.

Personal utility (if defined at all) is often characterized negatively and as something of a runner-up, applying to information which, though not directly clinically actionable, might nonetheless serve broader health or well-being ends, for example by encouraging protective behaviour change or helping to prepare one for future health impacts (Bunnik, Janssens, and Schermer 2015; Daack‐Hirsch et al. 2013; Foster, Mulvihill, and Sharp 2009). The purpose of this article is to propose and illustrate a means of conceptualizing one particular species of the personal utility of bioinformation: its potential value in an individual’s construction of her own identity. This conception is not intended to be exhaustive of all dimensions of personal utility. However, the aim is to provide a less negatively defined account of the personal value of some bioinformation and to explain why this can be seen as engaging information subjects’ interests in ways sufficiently significant to warrant attention in the ethical governance of their access to this information. Distinguishing it from some presentations of personal utility, identity-value is not framed here as an (inferior) alternative to clinical utility, but as a potentially important parallel consideration, and one that is not reducible merely to facilitating its subject-recipients’ autonomous choices about softer health matters.

The suggestion that particular kinds of personal bioinformation could be relevant to identity is not in itself new. For example, the European Court of Human Rights (ECtHR) has recognized an individual’s “right to know their [genetic] origins,” explicitly on identity grounds, under the Article 8 right to respect for private life (*Odièvre v France* 13 February 2003, [45]). Commercial direct-to-consumer (DTC) genomic testing services frequently appeal to potential customers’ interests in their identities when marketing their services, including supposed “ancestry tracing” analysis (Nordgren and Juengst 2009). Meanwhile, several academic commentaries examine, often from a critical perspective, the perceived significance of genetic or genomic findings and biometrics to identity (Ajana 2010; Hauskeller 2006; Hauskeller, Sturdy, and Tutton 2013; McGowan, Fishman, and Lambrix 2010; Nordgren and Juengst 2009).

However, the accounts of the connection between information and identity given in these contexts may be seen as displaying some, if not in every case all, of a number of limitations. First, the precise nature of the role that bioinformation plays in identity often remains opaque, sometimes compounded by ambiguity about which sense of the multifaceted concept of identity is being invoked. For example, the jurisprudence of the ECtHR displays some slippage between concerns associated with the (re)identification of numerically identical individuals and those associated with identity understood in the sense of self-characterization.^1^ Secondly, we might question the apparently exceptionalist, or at least narrow, focus on genetic or genomic information. Thirdly, these existing accounts may themselves express (or else voice concerns about others expressing) a contended bio-essentialist idea of identity (Hauskeller 2006; Marshall 2009; Nordgren and Juengst 2009).

Here my intention is to offer a means of conceptualizing and clarifying one particular kind of relationship between personal bioinformation and identity in order to address these limitations. The proposal is that a useful way of understanding this relationship is by coming to see the former as one of the tools that an individual might use to constitute the latter through the construction of her own self-narrative. I wish to suggest that this conception not only permits us to recognize the relevance to identity of a range of personal bioinformation beyond the genetic, it also accounts for the morally significant role of this information in our lives, hence our potentially considerable interests in accessing it. Moreover, it does so without depending on a bio-essentialist view of identity.

The following discussion will explore these claims further. This will include consideration of features of information that account for its identity-value, as well as those that could undermine this. The final section will consider the characteristics of informational transactions that help to determine whether these contribute in a constructive way to identity. Providing some means of making these distinctions is important if the account offered here is to be useful to practical questions concerning the governance of access to personal bioinformation. The conception of identity-value offered below will be instructive in contemporary deliberations about information subjects’ interests in a number of fields in which the generation of bioinformation could pose fresh challenges. These include biobanking and DTC genomic testing, as well as prospective and emerging fields such as in DTC neuroimaging services, diagnostic uses of implanted neurodevices, fetal genome testing, and current policy debates about what someone born following mitochondrial transfer should be told about their mitochondrial donor (Department of Health 2014, [2.23]).

## A Narrative Conception of Identity

Before proceeding to an example that illustrates the potential role of bioinformation in identity construction, it will be useful to say something about the narrative conception of identity underlying this. This will necessarily only be a brief sketch of a rich vein of philosophical thinking but will highlight the main features that inform the assertions made below.

The narrative view of personal identity on which the discussion here is based owes much to that developed by Marya Schechtman in *The Constitution of Selves* (1996), though related accounts may be found in the philosophy of Alasdair MacIntyre and Charles Taylor, amongst others (MacIntyre 1985; Taylor 1989).^2^ The narrative conception of identity addresses what Schechtman terms “the characterization question” of what makes someone the individual she is (1996, 73). The answer to this question, Schechtman argues, is that identity (in this sense) is constituted by a self-narrative that each of us constructs, albeit not in isolation but as part of, and shaped by, a life lived amongst others.

In suggesting here that we might usefully construe the role of bioinformation in identity in terms of providing a tool of narrative self-construction, the intention is not to deny that bioinformation could also be relevant to quite different numerical or taxonomic identity questions, such as those concerned with distinguishing or reidentifying discrete individuals, or establishing species boundaries (Hauskeller 2006). However, while the concern is with the personal utility of bioinformation, it is identity in the sense of self-characterization or self-conception as described by Schechtman that is most pertinent. This is because, according to this view, it is by reference to our self-narratives that we address questions about our self-interested concerns, such as whether we continue to exist as the same person, with enduring commitments and values, over time. Our self-narratives are the frameworks through which we filter and interpret our experiences. And it is by being integrated into our narratives that elements of our lives—for example, our motives and characteristics—count as *ours*. Our narratives are thus the means by which we make sense of our lives, and they help to determine those actions for which we are responsible (Schechtman 1996). It should be plain from this that being in a position to construct such a narrative has crucial normative consequences for the quality of our lives and our moral agency.

On Schechtman’s account, the construction of one’s narrative not only entails these normative consequences, but also normative conditions on which they depend. These she terms the “articulation” and “reality” constraints (1996, 114 and 119). These constraints mean that, although it is not supposed or required that we literally or perpetually relate our own self-stories, they must at least be relat*able* and intelligible to ourselves and to others. This requires, inter alia, that our narratives are both internally coherent (articulable) and broadly consistent with (the reality of) the world as experienced by others. Being in a position to construct an intelligible, coherent and realistic self-narrative, therefore, really matters as part of the richness of a fulfilling human existence. The proposal I wish to illustrate and defend here is that in many, if not all, circumstances, knowledge of our bodies, health, or biological relationships to others can make a sufficiently important contribution to our capacity to construct our own narrative identities, such that our access to the kinds of personal bioinformation that supply this knowledge also acquires ethical significance. And this significance is distinguishable from, and not reducible to, any clinical utility it may or may not have.

This, however, is where the proposal to be advanced here departs somewhat from accounts such as Schechtman’s. Her own account enumerates the constituents of self-narrative in terms of “traits, actions, experiences” and “characteristics” (Schechtman 1996, 77 and 94). While she does not explicitly preclude the incorporation of *biological* traits or experiences into self-narrative, neither does she acknowledge their possible role. Indeed, she seems to relegate the identity-relevance of the human body merely to the means by which others may (re)identify us, thus permitting the kinds of social interactions that contribute to self-building (Schechtman 1996). I wish to suggest that insights into and understanding of our biological selves in fact play a key role in our identities, and thus information that supports this is potentially of great value.

This proposal might seem to commit a category mistake because our bodily states, functions, or relationships are only “ours” in a passive, default sense, not because they are woven into our personal stories. Schechtman herself lodges this kind of objection when she observes that findings about the neurological correlates of behaviour should not be seen as threatening our own narrative explanations of our motivations, because these neuroscientific findings have no claim on being “prenarrative truth about the self” (Schechtman 2012, 75). The argument to be developed in this article, however, has no quarrel with this. It is not premised on the assumption that personal bioinformation reveals existing facts about an individual’s *identity*, but rather that it supplies knowledge of her biology or health which she may then judge to be relevant (or not) and choose to use (or not) in thinking about who she is and in developing her self-narrative accordingly. Bioinformation should, therefore, be seen as being valuable to identity in an instrumental rather than intrinsic sense. As I shall argue further below, this value is attributable to the fact that our narratives must, if they are to fulfil their normative interpretive and sense-making roles, reflect the inescapably materially embodied nature of human existence. The somewhat dualist perspective reflected in Schechtman’s account, in which self-constitution takes place in the mind, while the body is just a vehicle through which identity is enacted or located, seems strikingly to underplay the centrality of embodiment in our lives.^3^ The next section will provide a concrete example in order to illustrate the significance of this omission.

## An Example From Psychiatric Neuroimaging Research

Psychiatric neuroimaging research provides an illustration of one context in which potentially personally significant bioinformation is generated. This field of research analyses neuroimaging data gathered from participants to examine possible correlations between structural or functional brain features and outcomes of potential psychiatric interest, including diagnosis of disorders such as depression or schizophrenia, prediction of risk of illness, or responsiveness to treatment (Cooper et al. 2013). The example of psychiatric neuroimaging has been chosen here because it is a technology widely anticipated to be on the cusp of producing robust diagnostic or predictive findings relevant to individuals, yet it is also still subject to considerable doubts about its current capacities to provide reliable insights into our mental health (ibid). These features makes it useful for illustrating the potential for a field of biomedical research to generate rich and useful narrative tools, while also helping to demonstrate the ways in which the epistemic limitations of a technology like neuroimaging could significantly detract from this potential. That is, it captures both sides of the coin in respect of the ways in which the identity-value of personal bioinformation is contingent upon its strengths as a source of knowledge of our bodies, health, and biological relationships.

One example of recent psychiatric neuroimaging research is a study conducted with relatives of people with heritable bipolar disorder by the Division of Psychiatry at the University of Edinburgh, with the aim of investigating whether activation differences are evident in the brains of at-risk individuals prior to the onset of illness (Whalley et al. 2013).^4^ This study involved analysis of functional magnetic resonance imaging (fMRI) data from ninety-eight individuals known to be at genetic risk of developing such a disorder, and a control group of a further fifty-eight participants.^5^ At the start of the study none of the participants was diagnosed as having a mood disorder. At the time of the two year follow-up twenty of the at-risk participants had developed a major depressive disorder.

At the start of the study, all participants underwent an fMRI scan to capture data about their patterns of brain activation while they performed language processing exercises. The high-risk individuals who went on to develop a depressive disorder exhibited increased activation in a particular region of the brain (the bilateral insula cortex). This abnormal activation was not widely observed in either the high-risk individuals who remained well two years later, nor in the healthy control group. This supported the researchers’ hypothesis that the brains of those participants who developed a mood disorder would exhibit activation differences in the regions associated with depressive disorders. However, the incidence of false positives (findings of abnormal activation in participants who remained well at the two year follow-up) was around 25 per cent—the relevance of this to the identity-value of individual results will be considered further below. The researchers posited that their findings provided evidence of a possible predictive biomarker for mood disorder in those already at genetic risk, the clinical application of which warrants future research.

The study described here is intended to provide a concrete illustration to anchor further discussion; the implication is not that its findings exhibit unequivocal, unique, or exceptional identity relevance. Indeed one reason for choosing this (rather than more thoroughly traversed categories of bioinformation—such as those relating to genetic parentage (Kirkman 2003; Nuffield Council on Bioethics 2013; Ravelingien, Provoost, and Pennings 2015; Wilson 1997) or genetic disease risk (the potential identity relevance of which is widely discussed, if contested) (Hauskeller 2006; McGowan, Fishman, and Lambrix 2010; Nordgren and Juengst 2009) is to demonstrate that the arguments presented here are generalizable to a broader range of personal bioinformation. It is, however, also an interesting example in its own right. Unlike some other kinds of neuroimaging research, studies in this field can in principle produce results pertaining to individual participants, rather than just aggregate findings (Cooper et al. 2013). Psychiatric neuroimaging research represents a busy field in which there is considerable, if not universal, enthusiasm about the possibilities of clinical translation of its findings (Farah and Gillihan 2012; Kapur, Phillips, and Insel 2012; Reilly and McGuire 2013). As more studies are conducted, ethical and practical questions about whether participants’ interests in accessing individual results extend beyond clinical utility, which are increasingly being asked in other research fields, will only become more pressing. It is timely now, before predictive or diagnostic tests using neuroimaging reach the clinic, to interrogate any possible identity-related implications of disclosing test results that should be considered alongside their potential clinical utility. These implications include not only potentially constructive contributions, but also circumstances in which disclosure could be detrimental or simply not useful.

In the study described above, participants were informed that clinically significant incidental findings of structural brain abnormalities would be disclosed to their GPs (Division of Psychiatry, The University of Edinburgh 2008). However, there was no parallel policy to return the *intended* research results to participants, even if these were to provide probabilistic predictions or hypothesized explanation of their increased susceptibility to serious depressive disorders. This is noted here not to prejudge the appropriateness of such a policy but because it at least warrants some further examination from the perspective of inquiring whether such results could be valuable to participants on grounds of their identity-relevance. In order to address this question, the following paragraphs will first suggest several ways in which findings from a psychiatric neuroimaging study such as that described above could contribute constructively to the development of the subject-and-recipient’s self-narrative. Some ways in which this information might have less positive impacts on identity will also be reviewed. These inferences are based upon empirical literature reporting attitudes of patients, healthy individuals, and clinicians to (often hypothetical) scenarios in which predictions or diagnoses of mental illness are made or confirmed using neuroimaging data, as well as to other kinds of biologically-based explanations of cognitive or psychiatric disorders. Not all of these accounts make explicit references to identity, let alone self-narrative. Here I attempt to draw out how the attitudes reported might be relevant to narrative self-constitution.

For simplicity, the discussion in the next few paragraphs will assess possible positive and negative impacts on identity *as if* the findings from the study just described provide robust predictive or diagnostic information and therefore it would not be unreasonable for individual participants to treat them as reliable sources of knowledge about their (future) mental health. However, in reality, it is probably too soon to assume that research findings in the field of psychiatric neuroimaging are reliable in this way (Cooper et al. 2013; Farah and Gillihan 2012; Reilly and McGuire 2013). Moreover, the neurological basis of psychiatric disorders is itself not uncontested (Ramos 2012). Therefore, following an initial assessment of the kinds of features that constitute the identity-value of bioinformation, I will turn to consider how doubts about the capacity of psychiatric neuroimaging to provide reliable predictive or diagnostic results for individuals (or indeed about any bioinformation facing similar epistemic challenges) might affect its identity-value. First though, the following paragraphs will provide illustrations of how and why receipt of findings from a psychiatric neuroimaging study could potentially, mutatis mutandis, contribute in significant ways to an individual’s self-narrative.

On the basis of some accounts of experiences of living with mental illness, we might anticipate that for many recipients the most immediate identity-impact of results indicating an elevated risk of serious psychiatric illness would be fear that the onset of illness will be accompanied by a “loss of self” (Wisdom et al. 2008). However, receiving (reliable) information about one’s increased risk of psychiatric illness could, as well as heralding this threat, also be seen as offering a number of ways of countering the feared loss of self. It might provide the opportunity and encouragement to undertake protective measures such as behaviour changes or early interventions (where available and effective) (Borgelt, Buchman, and Illes 2011; Buchman et al. 2013; Gilbody, Sheldon, and House 2008; Marshall and Rathbone 2011). Just because these measures aim at improved health outcomes does not mean that the ends thus served are solely health-related. Without conceding a neuro-essentialist view of identity, we may still recognize the particularly intimate connections between a well-functioning brain, someone’s subjective experiences, and the integrity of her sense of self (Gillet 2008). The prevention of symptoms of serious mental health disorders may, therefore, itself be viewed as intimately connected to the protection of an individual’s relationship to her own self-narrative and identity.

Predictive findings could also serve a preparatory function by helping the subject-recipient to develop a revised self-narrative that supports her in making sense of and remaining resilient when confronted with the threat or onset of symptoms of mental illness. Carlos Novas, Nikolas Rose, and Joelle Abi-Rached have theorized that one response to new kinds of knowledge that biotechnologies such as genetic testing and neuroimaging make available is the adoption of a “somatic individuality,” which is characterized by awareness of being “at risk” (Novas and Rose 2001, 485 and 488; Rose 2007; Rose and Abi-Rached 2013). This mode of self-identification is presented as an empowering one in which the individual assumes responsibility for decisions about her health and her body by, for example, availing herself of the knowledge and opportunities to undertake preventative or therapeutic measures.

There are also indications from some studies that receiving an explanation of mental illness based upon neuroimaging findings could provide reassuring, objective validation of patients’ experiences, one which proves that they are not “just crazy” and lends “legitimacy and unarguability” to their health status (Buchman et al. 2013, 74; Cohn 2010) Even allowing for warranted concerns about whether such views attribute too much objectivity and authority to neuroimages,^6^ it might still be recognized that giving someone the opportunity to explain her symptoms in neurological terms could potentially support the development of a self-narrative that permits her to make sense of her experiences of illness, or even offer therapeutic benefits. For example, some psychiatric research has suggested that constructing self-narratives that incorporate acknowledgement of the reality of illness could support insight and, thus, recovery in patients with psychotic disorders (Roe and Davidson 2005; Wisdom et al. 2008).

In contrast to these suggestions of narrative accommodation, findings from psychiatric neuroimaging could alternatively facilitate a helpful distancing of the self from the disorder, in which the latter comes to be seen just as a feature of one’s brain as part of one’s body (Cohn 2010). One study suggests that one way in which this externalization might be beneficial is in mitigating patients’ sense of responsibility and self-blame for their illness (Illes et al. 2008). I would suggest that this separation of a psychiatric disorder from the self ought not to be read as indicating the irrelevance of the neurological findings to narrative construction. On the contrary, it seems reasonable to recognize that bioinformation is no less identity-significant for facilitating an individual’s *exclusion* of particular modes of biological self-definition from her narrative.

These indications of the potentially constructive role of bioinformation in narrative development notwithstanding, it is important to recognize that the same kind of information could instead be detrimental to the recipient’s sense of self. For example, one group of practitioners asked about the hypothetical clinical use of predictive neuroimaging expressed concern that this could disturb rather than empower patients’ sense of identity if they came to equate a disordered brain with a disordered self (Borgelt, Buchman, and Illes 2011). Indeed, we might heed warnings from research that found that simply informing participants that they carried a gene associated with increased risk of Alzheimer’s disease impacted negatively on both their own assessment of their memory and performance in memory tests (Lineweaver et al. 2013). Here bioinformation may still be seen as a narrative tool, but one that leads recipients to reinterpret who they are and their capacities in biologically deterministic ways that potentially undermine their wellbeing.

Negative identity impacts might equally arise from interpreting findings in ways that distance the source of psychiatric illness from the self. For example, one study found that, alongside reducing burdensome feelings of personal responsibility for depression, providing neurochemical explanations for this illness can also have the less desirable effects of increasing pessimism about recovery and scepticism about the effectiveness of psychosocial therapies (Deacon and Baird 2009). The irony here is that it is these kinds of therapies (as contrasted with psychopharmaceuticals) that “work on the self” and might therefore better lend themselves to the construction of therapeutic self-narratives (Rose and Abi-Rached 2013, 220). Similarly, while we might assume that biological explanations of psychiatric disorders would reduce the stigma of mental illness and associated ill-effects on self-conception, there is some evidence that neuroimaging-based explanations of disorders may make little difference to others’ attitudes (Cohn 2010), or (extrapolating from work on genetic explanations) could even exacerbate stigma if the disorders are thereby seen as more serious or intractable (Phelan 2005; Read 2007).

Before concluding this section, it is worth noting that it is not inevitable that all predictive neuroimaging findings will be received in ways that are unequivocally either beneficial or detrimental to the subject-recipient’s self-conception. There are some indications that individuals change their attitudes towards the identity-significance of facts about their brains depending on context or their current circumstances, or are simply ambivalent about whether these tell them anything about who they are (Dumit 2004; Pickersgill, Cunningham-Burley, and Martin 2011).

## Accounting for the Identity-Value of Personal Bioinformation

The examples of the possible impacts, interpretations, and uses of psychiatric neuroimaging findings outlined in the previous section are intended to be illustrative of the potential identity-significance of a wider range of personal bioinformation. In order to demonstrate this it will be useful to take stock of the kinds of narrative tools that bioinformation could provide, building upon but expanding beyond the specifics of psychiatric neuroimaging.

One central aspect of narrative self-constitution is that it provides the means of developing and thinking about *who* we are and the kinds of characteristics, values and commitments with which we choose to align our identities. To this end, then, bioinformation may be seen as providing a range of possible new or reconfigured modes of self-description: fresh means by which the recipient might classify herself (for example, as someone “at risk” or “a survivor” of a particular condition, or as “donor conceived”) (Hacking 1986; Hacking 1995). These descriptors might be freighted with particular significance because of how they affect the place of particular motivations, priorities or affiliations in our own stories. For example, coming to thinking of ourselves as “at risk” might lead us to give particular priority to our responsibilities for our own health as part of the commitments and projects that make up our narratives (Novas and Rose 2001). Or they could provide the impetus for new associations and relationships (perhaps with those with whom we share a disease risk or our donor siblings) or for joint endeavours such as patient activism, thus establishing fresh interdependences between our own narratives and those of others (Gibbon 2007). Or they might simply supply new ways of thinking about ourselves and some of the context and filters through which we interpret and order our experiences. None of this is to suggest, however, that any particular individual is obliged to use bioinformation in these ways. Crucially, on the account being developed here, bioinformation is a narrative tool that may be used, ignored, or reacted against, not a straightforward building block to be incorporated-as-given into one’s identity. The receipt of personal bioinformation could just as well support narrative developments that *resist* (re)definition in biological terms (for example, by refusing to be defined by one’s illness or one’s chromosomal sex).

However, according to the narrative-constituted account adopted here, identity is not just a random assemblage of selected self-descriptors. Our self-narratives provide the interpretive frameworks through which we make sense of who we are and our experiences and as such, their intelligibility and articulability comprise key normative features. Personal bioinformation, therefore, can fulfil a further kind of role by providing the kinds of explanations, connections or forewarnings that contribute to the intelligibility of our narratives, both in terms of their internal coherence and in relation to our own (and others’) experiences of the world . For example, bioinformation could supply explanations that help to fill (perhaps distressing) gaps or discrepancies between an individual’s existing beliefs about who and how she is and her subjective experiences; thereby permitting her to construct a story about her self and her life that makes sense of, and space for, these experiences. In a similar way, the information might help to disabuse her of unhelpful or misconceived interpretations of the causes of her experiences. Where the onset of illness or other physical or psychological changes have not yet impinged on someone’s experiences, bioinformation could also fulfil a pre-emptive role of an analogous kind, permitting the individual to prepare and plan for the changes to come and find ways to accommodate these within her developing narrative. We can, for example, imagine these roles being fulfilled by information about one’s genetic parentage that explains why one’s hereditary traits differ from those of other family members (Kirkman 2003), or by results from testing for genetic cancer risk which provide the opportunity for one to consider how this might impact on one’s identity as a potential parent (d’Agincourt-Canning 2006), as much as by indicators of mental health risk that could help one to make sense of the onset of affective or cognitive changes. Again, however, it is important not to overlook the possibility that bioinformation might not contribute to someone’s narrative in ways that she experiences as welcome or constructive. For example, this might be the case where findings are rendered only in probabilistic terms that only serve to increase uncertainty, or if they conflict with someone’s existing modes of self-understanding or the account of herself she sees projected in her future. I shall return to consider circumstances in which bioinformation might not be useful for identity formation further in the next section, but first there is a further step to be made in understanding this value itself. I wish to look beyond specific instances of the ways in which bioinformation might contribute to self-narrative, to examine the underlying explanation for the identity-value that these instances have in common.

When described in the less technology-specific terms above, it becomes possible to see how the instrumental role(s) of bioinformation in identity construction plays out beyond psychiatric neuroimaging findings, and how, for example, results from genomic screening, tests for specific disease risks, individual findings (anticipated or incidental) from health research, or information about one’s genetic origins might similarly provide useful narrative tools. The question remains, however, why having the opportunity to use personal bioinformation in the construction of one’s self-narrative is sufficiently important to warrant ethical attention. The answer to this, I wish to suggest, comprises two steps briefly introduced above. The first of these is that, on an account of narrative self-constitution such as Schechtman’s, being in a position to develop an intelligible and realistic self-narrative is a condition for achieving that which is valuable in an identity thus constituted. The second step diverges from Schechtman’s own account, because it rests on the assertion that the kinds of insights and understanding that individuals can derive from receipt of personal bioinformation contribute in an important way to securing this condition because of the inescapably materially embodied nature of our existence.

By the “embodied nature of existence” I mean that our biology and bodies exert what Stacy Alaimo and Susan Hekman characterize as “active” and “recalcitrant” forces upon our lives that serve to shape, enable, and place limits both on the nature of our experiences and on our capacities to define ourselves (Alaimo and Hekman 2008, 3–4). Examples such as pain, illness, and (dis)ability might come most readily to mind here, but our reproductive, cognitive, and affective capacities as well as the functioning of our autonomic systems, the observable markers of our social identities, and our biological relationships to others will also play a role. As Ian Hacking observes, however strongly inclined we are to the idea that we invent ourselves, we must recognize that we do so while “push[ing] our lives through a thicket in which the stern trunks of determinism are entangled in the twisting vines of chance” (Hacking 2004, 282). We do not need to follow Hacking in his language of determinism for his metaphor to remain apt. In pursuit of defining who we are, we will inevitably bump up against the realities and constraints of our material, biological selves. The suggestion I wish to make is that, by fulfilling the kinds of explanatory, preparatory, self-descriptive, or relation-building roles described above, personal bioinformation supplies us with the means to construct self-narratives that help us to negotiate some, though probably not all, of our recalcitrant materiality. It alerts us to the whereabouts of some of the “stern trunks” and “twisting vines” and thus to live out identities that support us in anticipating their impact, embracing them, or navigating around them.

The assertion here, then, is not merely that it is satisfying or interesting if our self-stories include plotlines that involve features of our embodied state or even that they are really likely to do so because those features are prevalent in our lived experience, but the stronger claim that the incorporation of these is key to meeting the conditions of articulation and reality that Schechtman imposes on a robust identity-constituting narrative. Thus access to the kinds of information that facilitate the construction of these plotlines could be key to realizing the value inherent in developing and understanding and enacting who we are. Given that we lead inescapably embodied lives, a narrative that is developed with knowledge of, and insight into, our own biology and health will be one that makes sense to ourselves and to others when confronted with the reality and vagaries of our materially embodied existence. It will also be one that, in turn, helps us to interpret and make sense of this reality and to negotiate the experiences with which it presents us. In doing so, personal bioinformation will contribute to the construction of a rich and intelligible self-narrative of a kind that retains its integrity, supports our enduring values and commitments, underpins our moral agency, and supplies interpretive perspective on the world and thus comprises an important part of a full and flourishing existence. This accounts for what, I wish to argue, is our ethically considerable interest in (at least being given the option of) accessing this information.

One important part of the value of a self-narrative constructed in the light of knowledge of one’s embodied existence derives from the intimate connection between such a narrative and personal autonomy, where autonomy is understood as the capacity for critical reflection upon and evaluation of one’s motives (Dworkin 1988). An individual may be seen as exercising this capacity in her assessment and selection of the elements from which she constructs her own narrative. Moreover, and more fundamentally, this self-narrative in turn supplies the source of the considered desires, beliefs, and values that provide motivations for an individual’s autonomous actions—those actions that are in Schechtman’s terms “quite solidly hers”—and in which, on many conceptions, autonomy itself is rooted (Schechtman 1996, 81; Dworkin 1988; Watson 2003). Beliefs about our bodies, health, and biological relationships, insofar as they supply the basis for the many of our personal projects, values, and interpersonal commitments are likely to supply much of the material of autonomy thus conceived. Furthermore, a self-narrative informed by knowledge of our biological selves may help to provide some of the foundation for, and critical perspective upon, a system of considered motives that maintains their integrity and coherence when confronted with the realities of embodied existence. It might be objected here that narrative strands derived from personal bioinformation are, on the contrary, antithetical to autonomy, originating as they do in biological factors that are externally imposed rather than reflected upon and chosen. However, as John Christman (2001) observes, while we may be heteronomous with respect to those aspects of our physical selves from which we feel alienated, a conception of autonomy that requires total substantive independence from our biology and physicality not only sets an unfeasibly high bar for achieving autonomy, but also fails to capture the important role that embodiment plays in shaping who we are. Insofar as autonomy provides the “means to our working out our projects in the world,” permitting us to realize our goals and exercise our moral agency, the means and opportunities to develop reflective capacities and to act on these can be regarded as intrinsically valuable parts of a fulfilling human existence (Young 1982, 43). Therefore, being in a position to construct a coherent narrative, which draws on knowledge of one’s own biology for its constituent elements and the evaluations we apply to these, is something that supports autonomy and thus contributes to the meaning and richness of one’s life.

Whilst there is indeed a close connection between the construction of an intelligible self-narrative and autonomy, this chiefly pertains to autonomy understood in a thick sense of a global capacity of a whole person. Where autonomy is understood in a thinner sense (one prevalent in many bioethical texts), as the context-relative state of being appropriately equipped to make a discrete practical choices or as synonymous with informed consent (O’Neill 2002), the utility of personal bioinformation is reduced to nothing more than its capacity to inform particular decisions. Some of the value of the narrative contribution of bioinformation, as proposed here, might indeed lie in supporting practical decision-making. But the suggestion is that its significance also extends far beyond its imminent action-guiding potential and may indeed endure despite not being applicable to any immediate decisions.

The importance of a self-narrative informed by personal bioinformation is also not reducible solely to its role in supporting autonomy. According to a conception such as Gerald Dworkin’s, autonomy is significant because of the role it plays in personal agency. Autonomy thus conceived should be seen as only one of the valuable capacities that is underpinned by a coherent self-narrative. As Dworkin himself notes, “[a]utonomy is important, but so is the capacity for sympathetic identification with others, the capacity to reason prudentially, or the virtue of integrity” (1988, 32). The value of a narrative that makes sense in the context of embodied existence is located as much in its power to effect changes in someone’s view of herself and of others—her values, allegiances, and interpretive perspective upon the world which might never, or at least not necessarily, lead to specific actions.

It should now be apparent that, on the account offered here, the identity-value of personal bioinformation is not just clinical utility under another name. It is not reducible merely to a quality that facilitates discrete, autonomous, health-related decisions. The identity-value of bioinformation can obtain where clinical utility does not, or in contexts where clinical choices do not arise. However, neither is identity-value merely an alternative to clinical identity. As previously mentioned, personal utility is often invoked as an explanation of the value that information might retain even when the criteria for clinical utility are not met—where utility is rendered instead in terms of softer health or well-being outcomes. The present account of identity-value is importantly distinct from such accounts of personal utility. The argument offered here is that an individual’s identity-related interests in bioinformation warrant ethical attention both alongside and independently of her clinical and wider health interests.

Before moving on to consider the kinds of factors that might influence whether particular instances of bioinformation are useful for the purposes of self-conception, I wish to head off one erroneous inference that might be drawn from asserting the identity-value of bioinformation in general: that this necessarily entails a bio-essentialist view of identity. It does not. Conceptions of personal identity as self-created are often contrasted with those in which it is seen as essential (Marshall 2009; Nordgren and Juengst 2009). Accounts that propose a significant role for knowledge of one’s biology are often assumed to fall in the latter camp, where information is framed a means of discovering a pre-existing essence. As such, they can be seen as objectionable for denigrating the choices of those who characterize themselves in ways that diverge from their biology, for example, by identifying as transgender or embracing their social family as their origins story. However, as should be clear by now, on the account offered here, personal bioinformation is not valuable because it reveals who we already are but because (with some provisos to which I shall return below) it provides knowledge of our embodied states that is potentially useful in developing who we are. Particular kinds of bioinformation are not intrinsically identity-relevant, but only insofar as they serve the ends of helping someone to construct an articulable, intelligible, and realistic self-narrative. It is not necessarily a threat to this intelligibility if someone responds to the receipt of bioinformation by (re)asserting their identification with non-biological aspects of their lives such as their chosen family or gender. Nothing in the present account entails that *bio*information provides the only tools for a coherent, comprehensible, and satisfying self-conception; our narratives will inevitably also be woven from strands that have nothing to do with our biology.

## Factors That Could Detract From, or Enhance, Identity-Value

Attending to the reasons offered here for the potential identity-value of personal bioinformation brings to light two corollary factors that might impact upon the capacity of information to provide truly useful narrative tools. The first of these concerns the empirical robustness of the information and its associated capacity to provide relevant knowledge of our embodied states. The second relates to the crucial part played by interpretive context in how the information is received and utilized. Each of these will be considered in turn below.

If the identity-value of personal bioinformation is located in its capacity to help someone build a narrative that is in tune with an embodied existence, then this has one particularly important consequence when it comes to assessing which kinds of information are suited to fulfilling this role. I wish to suggest that personal bioinformation will only provide a useful tool of identity construction to the extent that it (including the context in which it is conveyed—the importance of which is unpacked further below) supports its subject-recipient in forming *reliable* beliefs about the nature of her own body, biology or health. This, I wish to suggest, means that the bioinformation that contributes to our narratives in useful ways must at least be empirically robust. This may be seen as taking Schechtman’s reality constraint one step further than she herself does, though with a parallel justification. Schechtman’s reality constraint requires that our narratives are reasonably consistent with the world as experienced by others, because this is a condition for our being able to function in *social* contexts (Schechtman 1996, 119). On the account developed here, our narratives need also to be reasonably consistent with our *biological* reality, because only then are they likely to support us effectively in making sense of and navigating our experiences of embodied existence, and themselves remain intelligible in light of these.

This need not entail a strongly realist attachment to the scientific “truth” of all potentially valuable bioinformation. It is sufficient that this information is consistent with the phenomenological world, that it leads the recipient to form beliefs about her body, health and biological relationships amongst which, in Bas van Fraassen’s phrase, her actual and potential experiences can “find a home” (van Fraassen 1980, 86). This is because what matters is that personal bioinformation provides the recipient with sufficiently reliable knowledge and understanding of the past, present, or probable future nature of her embodied state to fulfil the kinds of explanatory, predictive, or descriptive functions described above, such that she does not end up constructing a self-narrative that grates against, ceases to make sense, or causes her to stub her toes painfully when confronted by the realities of her embodied existence.

In order to illustrate the dependence of the identity-value of bioinformation upon its capacity to underpin reliable beliefs, I will return to the example of psychiatric neuroimaging research findings and the deferred question of whether their potential positive narrative contributions (as previously described) are likely to be realized in practice. Despite the assumption hitherto made for simplicity’s sake—that the kind of predictive neuroimaging research findings discussed could function as useful narrative tools in various ways—there are several grounds for questioning this assumption. First it might be objected that a fundamental obstacle to their value is that knowledge of our brain functions is not the same as knowledge of mental illness or its impact upon our own lives (Glannon 2009; Ramos 2012). Even if one does not subscribe to wholesale scepticism about the role of the brain in mental illness, one might nevertheless recognize that to understand mental disorders we need to look not to the brain alone, but to its interaction with its bodily, cultural, and social environment—to the functions of what Walter Glannon terms the “embodied and embedded mind” (2009, 321). A related objection is that findings such as the outputs of fMRI are just dry, disembodied biomedical data and as such fail to capture the individual phenomenology of illness and, therefore, are incapable of fulfilling the kinds of descriptive or explanatory narrative roles which would on the present account justify their identity significance (Mazanderani, Locock, and Powell 2013). However, the account of identity-value offered in the previous sections does not rely on the claim that bioinformation provides the complete story of someone’s experiences of embodiment and illness, with all the personal nuances this entails. Neuroimaging findings taken in isolation are indeed unlikely to equip someone with everything she needs to construct an identity capable of navigating the lived and felt realities of mental ill-health, but this does not mean that they could never be of use to her in helping to fill gaps, frame experiences, or seed affiliations. Whether biomedical findings are in fact too abstract or impersonal will, to a considerable extent, depend on how they are conveyed and the interpretative support provided as part of this. I wish to suggest that the legitimacy of the above objections (excepting those premised on thoroughgoing scepticism about the brain’s role in mental illness) can be acknowledged without relinquishing the proposition that information about brain function or structure could provide valuable narrative tools.

Having said this, I would maintain that the possible value of this instrumental, contributory role of neuroimaging findings in self-conception is not unassailable, but contingent upon the reliability and predictive strength of the information in question. The example discussed above is more vulnerable to this second kind of concern than the previous objections. There remain legitimate reservations about the ability of current psychiatric neuroimaging techniques to provide robust, predictive findings about an individual’s health. This is attributable to, amongst other factors, a lack of standardisation in imaging methodologies (Farah and Gillihan 2012) and doubts that current psychiatric diagnostic categories align neatly with structural or functional neurological biomarkers (Ramos 2012). There are related concerns that results from psychiatric neuroimaging do not yet tend to exhibit sufficient sensitivity (to differences between individuals’ brains) or specificity (to different mental health conditions) (Farah and Gillihan 2012). These kinds of limitations are manifest in the specific study cited above, in which the incidence of false positive findings was around 25 per cent. This degree of error, I would suggest, is material to our assessments of the information’s potential identity-value.

The intention here is not to question the quality of the methodology used in the study described. Rather, it is to highlight that findings from psychiatric neuroimaging research such as this will only contribute usefully to narrative construction insofar as they are reliably predictive and explanatory of an individual participant’s future risks of developing a depressive disorder. There is little value, and perhaps even a real risk of harm, in someone constructing and living-out an identity that acknowledges, accommodates, and prepares for the onset of a serious depressive disorder if she is not in fact at increased risk, or indeed in being falsely reassured if she is at risk.^7^ While I have suggested that research participants could have ethically relevant interests in (at least being offered) access to individual research results as a tool of identity construction, this interest is unlikely to obtain when these results would not provide them with useful insights into or understanding of the world in order to construct a narrative that remains intelligible as their experiences of the world unfold. Given the relatively high incidence of false positives, which introduces a level of uncertainty about whether any particular participant has an elevated risk of developing depression, the policy of not disclosing individual research results to participants in the study in question looks sound, at least as far as protecting participants against misleading identity impacts are concerned.

As previously noted, the example of psychiatric neuroimaging is useful in capturing both sides of the account of identity-value offered here, illustrating the potential for biomedical research to generate information that could be useful for constructing an intelligible story of an embodied existence, while in practice still exhibiting features that constrain the actualization of this kind of value. Concerns about the epistemic limits of particular categories of bioinformation apply not only to findings from pre-clinical research studies, which inevitably involve some degree of uncertainty, but extend also to other contexts such as the outcomes of some clinical tests for some genetic disease risks where the results themselves are rendered in probabilistic terms (Atkins and Panegyres 2011). The extent to which bioinformation provides a “good enough” chart of the submerged trunks of someone’s embodied reality such that it will provide a reliably useful tool for narrative construction is clearly something that will admit of degrees, depending on a number of factors including the seriousness or significance with which that particular aspect of her health or biology is imbued. Some level of uncertainty may be inherent to the probabilistic nature of the results currently achievable in particular biomedical fields and (perhaps accompanied by appropriate contextualisation and counselling) need not necessarily obviate all identity-value.

However, it is worth noting that sound findings that convey uncertainty about an individual’s future health occupy one end of a spectrum, at the other end of which are those which are frankly unsupported by evidence. If the identity-value of results from well-conducted research is questionable, then this doubt applies with even greater force to results generated from, for example, poor or inappropriate observational or analytical methods, or overambitious extrapolations from observed data, as may well be the case in some dubious DTC services prematurely offering putatively “diagnostic” neuroimaging (Alpert 2012; Borgelt, Buchman, and Illes 2012), or genetic ancestry testing (Royal et al. 2010). Concerns about the disvalue, or actual harm, of disclosing empirically unsound information of these kinds would, I suggest, extend even to circumstances in which the information-subject herself really desires access to it.

It is important to recognize the scope of the claim being made in this section. It is not that all empirically robust personal bioinformation is relevant and of value to its subject-recipient’s self-narrative, that will depend as much on the story she wishes to tell about herself. Empirical robustness, I wish to suggest, is a necessary, but not a sufficient condition for identity-value. Some commentators have suggested that a similar condition of analytic or clinical validity should apply when recognizing the personal utility of genomic test results or research findings (Bunnik, Janssens, and Schermer 2015; Wolf et al. 2012). However, in those contexts it is not always clear why the imposition of such conditions is appropriate if the utility of the findings is indeed *personal*. Here I hope to have provided an explanation why at least one species of personal utility (its personal nature notwithstanding) is conditional on the objective criterion of empirical robustness.

The preceding discussion outlines the most fundamental factors influencing information’s identity-value, but does not yet provide anything like a definitive answer to the question of when personal bioinformation should be disclosed to the individual to whom it pertains. Just because information is empirically robust does not mean that the identity-related interests of the individual(s) to whom it applies will automatically be served by accessing it. It is important not to lose sight of the possibility that, in some cases, receipt of bioinformation could be disruptive rather than welcome. New—particularly unexpected and unsought—bioinformation might be distressing where it contradicts valued aspects of the recipient’s existing narrative, for example, as in some revelations of genetic parentage (Kirkman 2003). Alternatively, as illustrated above, it could feed biologically reductive self-conceptions to the detriment of her quality of life, for example, by encouraging denigratory self-perceptions or a fatalistic passivity. This need not be taken as indicating the identity-*ir*relevance of information received in these ways (indeed it signals the importance of attending to potential identity impacts of all shades when assessing ethical disclosure practices) but it certainly signals a lack of unequivocally positive identity impacts.

For example, tests currently available for detecting the BRCA1 and BRAC2 genetic mutations offer clinically valid results indicating that carriers of these mutations have significantly increased lifetime risks of developing breast and ovarian cancer (albeit probabilistic results rather than 100 per cent certainty).^8^ However, simply knowing that these tests can accurately identify an individual’s clinical status will not on its own tell professionals whether disclosing results to the tested individual will, on balance, have a positively constructive impact on her self-conception. The relevant considerations that professionals responsible for information disclosure would need to take into account in deciding whether to provide access to personal bioinformation on identity-related grounds are, first, whether it would provide the individual with sufficiently reliable knowledge of her aspects of biology or health to enable her to navigate the lived reality of these aspects, and second, whether there are reasons to anticipate that the information would have significantly negative impacts on her sense of self. While some individuals may seek BRCA1/2 tests in order to be in a position to take potentially protective measures and to gain some understanding of and control over their future experiences, others may eschew testing, fearing that the results could threaten their existing narratives by, for example, making them feel betrayed by their bodies or changing the dynamics of their family relationships (d’Agincourt-Canning 2006; Esplen et al. 2009).

Practical applications of the present account of identity-significance in healthcare, screening, or research contexts would need to take seriously the risks associated with the possible negative identity-impacts of disclosure. Yet professionals are unlikely to be able to discern *a priori* when the benefits of equipping someone to develop a self-narrative that remains intelligible in light of her experiences of biological “reality” might fail to outweigh any accompanying risks of distress or unhelpful bio-deterministic thinking in her particular case. This is not, however, a challenge unique to considerations of identity-value. Any recommendations regarding the disclosure of findings that seek to make space for recognizing their personal utility, beyond the rigid and objectively defined confines of clinical utility, are likely to encounter similar problems in anticipating when new knowledge would (not) be welcomed by an individual.

These considerations suggest that there will rarely be circumstances in which the unexpected imposition of unsought information would be justified on the grounds of furthering someone else’s project of self-conception. Indeed, they would counsel a cautious and attentive approach even where the information is sought by the individual, so as to ensure (as far as possible) that it is received in a way that enables her to appreciate its epistemic limits, and averts the kinds of interpretations that could be corrosive to a resilient or coherent sense of self. Such nuanced judgements about identity impacts do not have to be made, and indeed cannot be reached, in abstract. In practice, professionals would need to attend to individual-specific factors determining how the information would be received, such as the recipient’s personal values and circumstances and her capacities to take in, understand, and perhaps act upon what she is being told. While this could be resource-intensive, the professional responsibilities implied—to work to a greater extent in partnership with the patient or research participant—are in step with contemporary recommendations regarding informed consent in clinical and research settings. In that context it is increasingly common to encounter arguments that patients’ and participants’ consent should be a supported, relational, longitudinal process, reached in partnership with professionals, rather than a discrete one-off event (Laurie and Postan 2013; Maclean 2009). In the case of BRCA1/2 testing, this relational process could draw upon existing “self-concept” impact tools that could assist practitioners in delivering care that takes into account the possible effects on an individual’s sense of identity (Esplen et al. 2009).

The possibility of adopting these kinds of more interactive and insight-driven approaches notwithstanding, attending to identity-related interests will not eliminate familiar challenges arising from, inter alia, how to balance the tested individual’s identity interests against competing interests (for example those of blood-relatives whose own carrier status could be revealed), or how to manage the so-called “right not to know.” However, looking beyond the traditional concerns with autonomy and privacy, and taking into account identity interests, could help to shine fresh light into the nature of the values potentially at work in these hoary dilemmas. For example, Graeme Laurie suggests that the right not to know genetic information about oneself may be construed in terms of the interest in protecting one’s spatial privacy (Laurie 2002). Recognizing the potential identity-significance of genetic test results illuminates what might be seen as a key aspect of the value of spatial privacy: the protection of an environment in which an individual can construct her own self-narrative without unsought and unwelcome constraints.

To a great extent, however, few of us are free to construct our own self-stories entirely on our own terms. This is due in part to the fact that the perceived identity-value of particular categories of bioinformation is perhaps inevitably shaped by the kinds of accounts that prevail in the cultural and interpretive communities to which we belong. Social scientists have noted the pervasiveness of neuro-explanations amongst contemporary popular accounts of why we are who we are (Choudhury, Nagel, and Slaby 2009; Rose and Abi-Rached 2013). The abilities of neuroimaging, perhaps particularly fMRI, to provide direct insights into our identities are often subject to considerable levels of oversimplification and hyperbole when, for example, reported in non-specialist media or used by DTC neuroimaging companies in their marketing materials (Racine, Bar-Ilan, and Illes 2005; Racine, van der Loos, and Illes 2007).^9^ While such misrepresentations need not necessarily detract from the utility of more robust neuro-findings as narrative tools, it does point to the possible need to temper perceptions of their significance, particularly where (as suggested above) neuro-essentialist interpretations could be counter-therapeutic. Each of the concerns sketched above—from the risk of reductionist conflation of representations of our brain functions with our mental states, to doubts about the sensitivity of psychiatric research results, and awareness of the influence of hyperbolic claims about the capacities of particular kinds of bioinformation to provide insights into who we are—usefully highlight how important it is likely to be for those responsible for disclosure of personal bioinformation also to provide context for this information. That is, to provide context that helps the recipient herself to appreciate the possible limitations of particular findings or results as useful means of self-conception. This brings the discussion to one final important factor that could influence the potential identity-value of personal bioinformation. It is crucial to recognize that the utilization of information about our biology and health in our self-narratives is an interpretive undertaking and, furthermore, that information itself is not a discrete, inert entity that remains unchanged from one transaction to the next, but rather acquires its meaning and significance from the contexts in which it is conveyed and interpreted (Taylor 2012).

The malleability of the meaning and utility of information is particularly significant given that, as illustrated above, it is not inevitable that all the impacts of receiving bioinformation affect our identities in positive ways. This introduces important opportunities for, and indeed responsibilities upon, the professionals tasked with disclosure to take steps to support constructive, and minimize reductive or distressing, interpretations by those to whom they disclose. These could include, for example, explaining the limits of a research finding’s predictive or explanatory capacities, contextualizing test results for disease susceptibility with statistics on population-wide incidence, or providing links to patient support groups. It will also mean attending to exactly what kind and extent of information would best meet the explanatory, predictive, (re)descriptive or relationship-building ends of effective narrative tools. For example, it will be important to ask whether a donor-conceived individual’s identity-related interest in “knowing her genetic origins” will be adequately met by basic information about her gamete donor’s name, or whether it would also include the means of contacting the donor, or whether it is the story of her parents’ family-making choices that really matter to her (Blauwhoff 2008; Ravelingien, Provoost, and Pennings 2015). Although, up to this point, the present discussion has been framed in terms of the identity-value or disclosure of “the” personal bioinformation, it is plain that that which is valuable is not a neatly bounded package, but something that extends into the manner and context of its transmission to the individual. Questions about the “what” and the “how” of disclosure are, therefore, just as important as the “whether.”

Similar concerns about the need to contextualize bioinformation to avoid misconstrual are regularly raised in relation to the return of results from DTC genomic testing services without the intercession of advice from a suitably qualified clinician (Nuffield Council on Bioethics 2010). The present context, however, differs in a key way from those in which it is *clinical* utility of findings that requires contextualizing. While biomedical professionals who generate or disclose personal bioinformation may have the appropriate expertise to interpret its clinical validity, we might question the limits of their legitimate epistemic authority to interpret and help determine matters of its identity-significance. Here the worry is not merely that, in contrast to their clinical skills, these professionals might be ill-equipped to anticipate or support subjects’ identity interests. Instead, the concern is that despite this deficit they are nevertheless in a position to exercise disproportionate influence on how we define ourselves by shaping the availability, meaning, and perceived significance of the informational tools that are available for this task. This could operate at the level of the individual disclosure, in which a specific type of bioinformation, such as a disease diagnosis, is communicated in a fashion that emphasizes the gravity of its personal significance. It could also operate at a broader level through activities that contribute to shaping community-wide norms relating to the perceived relevance of a category of information as a mode of self-identification (Butler 2005), as in the current fevered environment in which neuro-explanations for all manner of human experiences are popular currency (O’Connor, Rees, and Joffe 2012). Either of these kinds of interpretive-loading could be seen as foreclosing an individual’s own perception of which bioinformation is (not) relevant to her identity.

To some extent the potential for external interpretive influences upon our perceptions of identity-significance is an inevitable (and not necessarily malign) consequence of the fact that we construct our self-narratives within communities and traditions, influenced by the ways that others define us (Hauskeller, Sturdy, and Tutton 2013; MacIntyre 1985). Furthermore, it would be a mistake to regard the individual as passive in this respect. As Hacking observes, by identifying with and living with a particular mode of self-identification, individuals themselves are active in shaping its meaning and scope (Hacking 1995). Nevertheless, there is, undoubtedly, a delicate balance to be struck in equipping those who generate and communicate personal bioinformation as part of their professional roles to do so in ways that are supportive of the kind of identity-development that enhances rather than undermines well-being, without thereby ceding to them too great an influence over determinations of identity-significance, when it is properly the subject not the producer of the information who should decide what story she wishes to tell about who she is.

## Concluding Remarks

Discussions of the personal utility and identity-impacts of findings generated in healthcare, screening services, and research are increasingly in evidence in the bioethical literature. However, often lacking from these discussions is a clear analysis of the basis of the personal or identity-significance of this kind of information. If these discussions are to have useful practical application, it is important to be able to characterize the nature of the value in question, because only then can we assess the nature, scope, and weight of the personal interests involved.

It has been suggested here that one aspect of the personal value of bioinformation may be explained in terms of the role it could play as a predictive, explanatory, descriptive, or relational tool in an individual’s construction of the narrative that constitutes her identity. The instrumental, rather than essential, nature of the potential contribution of bioinformation to identity should not, however, be taken as an indication of its triviality or dispensability. On the contrary, because of the inescapably embodied nature of our existence, personal bioinformation supports our capacities to construct identities that permit us to make sense of and navigate our experiences of this kind of existence and to remain intelligible in light of these. Moreover, because the construction of an intelligible self-narrative plays a central role in an individual’s identity as a social actor and moral agent, factors that impinge upon her capacity to undertake this construction engage her interests in ethically considerable ways—hence the factors determining her (in)access to and interpretations of the kinds of bioinformation that could fulfil this role are themselves ethically significant.

The instrumental role of bioinformation in our self-narratives helps to explain both its value and the kinds of factors that might undermine or enhance this value. However, individual preferences, dispositions, or capacities that might contribute to determining whether particular informational transactions will fulfil this role in positive ways present practical challenges to anticipating when and how information disclosure would be of unequivocal benefit to a particular individual. The account offered here does not pretend to provide answers to all the practical challenges that might accompany decisions about information disclosure. However, what it aims to have done is to make a case that the identity-value warrants serious ethical attention, alongside considerations of clinical and personal utility, in the context of governing access to personal bioinformation generated in healthcare, health research, screening, and assisted reproductive services.

## Footnotes

1 Concerns about numerical identity relate to questions about what determines whether someone or something is one and the same person or entity. When applied to people, these concerns often manifest in questions about the criteria necessary to identify someone as (remaining) the same individual, perhaps despite qualitative changes. Marya Schechtman, amongst others, has argued that it is a mistake to conflate questions about re-identification with those about self-characterization, which are instead concerned with the characteristics that make someone the kind of individual she is (Schechtman 1996). One example of an instance in which these two sense of identity are invoked may be seen in the ECtHR’s judgment in *Daróczy v Hungary* (1 July 2008) concerning the applicant’s interests in retaining the name by which she had been known throughout her married life. The judgment shifts between issues relating to the utility of a name as a means of (numerical) identification for administrative purposes and the role of someone’s name in her own self-definition.

2 Other philosophers who are closely associated with somewhat different narrative accounts of selfhood include Paul Ricoeur (1992) and Daniel Dennett (1991). Narrative theories of identity are not limited to philosophical inquiry, but extend also into sociology (Giddens 1991) and psychology (Gergen and Gergen 1988).

3 Catriona Mackenzie has made a similar critique of Schechtman’s lack of acknowledgement of the importance of our embodiment in her conception of narrative self-constitution, though without the aim of emphasizing the consequent role of bioinformation in this (Mackenzie 2009). Schechtman, however, is not alone in presenting a disembodied conception of narrative identity; Anthony Giddens’s account is subject to similar charges (see, for example, Witz 2000).

4 First-degree relatives of individuals with bipolar disorder are more than ten times more likely to develop bipolar disorder, and are three times more likely to develop a major depressive disorder, than the general population (Whalley et al. 2013)

5 fMRI is a non-invasive imaging technique that provides an indirect measure of brain activity relative to particular experimental tasks by detecting changing flows of oxygenated blood in regions of the brain.

6 The concern here is that patients might infer too much from the apparent objectivity of neuroimages, which are not in fact literal photographic representations of the brain depicting the location of mental illness, but rather artefacts constructed from complex statistical analysis (Roskies 2008)

7 For commentators inclined to wholesale scepticism about the role of brain function in mental illness, concerns about the possible harm, or at least disutility, of using neuroimaging findings to reify increased susceptibility to depression within one’s self-conception are likely to go even deeper.

8 For example, a previously unaffected woman who tests positive for the BRCA1 gene mutation has a 60–90 per cent lifetime risk of developing breast cancer (The Royal Marsden, NHS Foundation Trust 2013).

9 This is not necessarily a wholly new phenomenon, Borck notes the enthusiasm with which the “electric epistemology” of electroencephalography (EEG) was embraced in Weimar Germany (Borck 2001, abstract).
